# An Experimental History of the Materia Medica, or of the Natural and Artificial Substances Made Use of in Medicine: Containing a Compendious View of Their Natural History, an Account of Their Pharmaceutic Properties, and an Estimate of Their Medicinal Powers, so Far as They Can Be Ascertained by Experience, or by Rational Induction from Their Sensible Qualities

**Published:** 1785

**Authors:** 


VI. *An experimental History of the Materia Medica, or of the natural and artificial substances made use of in Medicine: containing a compendious view of their natural history, an account of their pharmaceutic properties, and an estimate of their medicinal powers, so far as they can be ascertained by experience, or by rational induction from their sensible qualities. By* William Lewis, *M. B. F. R. S. The third edition, with numerous additions and corrections. By*

 John

John Aikin. 4to. Johnfon, *London*, 1784. 691 pages.

A Confiderable time having elapfed fince the publication of Dr. Lewis's valuable work, it was thought right, now that a new edition was called for, to procure fuch additions and corrections, as later improvements in phyfic and natural hiftory had rendered neceffary. For this purpofe, it was put into the hands of the prefent editor, who is already well known to the medical reader, as the author of feveral ingenious writings, and who has, in the following manner, endeavoured to fulfil the intention of the publifhers.

To every vegetable article, he has added the Linnæan name. He has likewife corrected all the references to the Edinburgh Pharmacopeia, both in the catalogue of fimples, and the officinal preparations, by the laft edition of that work; and he has added feveral new articles *,

* The new articles are Aer fixus, Cardamine, Columbo, Filix mas, Flammula Jovis, Geoffræa Jamaicenfis, Lichen Iflandicus, Lobelia Syphilitica, Oenanthe Crocata, Peruvianus Cortex Ruber, Cinchona Carribæa, Pulfatilla Nigricans, Quaffia, Radix Lopeziana, Rhododendron Chryfanthemum, Spigelia, Stramonium, Viola Tricolor, Winteranus Cortex, Aconitum Napellus, Curfuta, Hippocaftanum.

among which are all the new medicines received into the prefent Edinburgh catalogue, and fuch others as have been recommended upon apparently fufficient grounds by late writers.

From the fame fources, and alfo from fome marginal notes in Dr. Lewis's own copy of his work, he has derived many additional facts and obfervations relative to former articles. All the additional matter is diftinguifhed by an afterifk prefixed.

At the fame time that Dr. Aikin has fo freely added to this work, he has not thought it allowable to omit any thing; though fome of the articles are now expunged from the catalogues of Materia Medica, and the ftate of Medical Theory has undergone confiderable changes fince the author wrote. It is ftill, in every refpect, Dr. Lewis's entire work.

